# The mitochondrial genome of two long-distance migratory shorebirds: the Hudsonian godwit (*Limosa haemastica*) and the Red knot (*Calidris canutus*)

**DOI:** 10.1080/23802359.2020.1827997

**Published:** 2020-10-12

**Authors:** Camila Gherardi-Fuentes, Juan G. Navedo, Claudio Verdugo

**Affiliations:** a Bird Ecology Lab, Instituto de Ciencias Marinas y Limnológicas, Universidad Austral de Chile, Valdivia, Chile; b Programa de Doctorado en Biología Marina, Instituto de Ciencias Marinas y Limnológicas, Universidad Austral de Chile, Valdivia, Chile; c Facultad de Ciencias, Estación Experimental Quempillén (Chiloé), Universidad Austral de Chile, Valdivia, Chile; d Facultad de Ciencias Veterinarias, Ecology and Evolution of Infectious Diseases Lab, Instituto de Patología Animal, Universidad Austral de Chile, Valdivia, Chile

**Keywords:** *Limosa haemastica*, *Calidris canutus*, shorebirds

## Abstract

We report the mitochondrial genome sequences of two migratory shorebirds, the Hudsonian godwit (*Limosa haemastica*) and the Red knot (*Calidris canutus*) obtained through shotgun sequencing. The mitogenome is of 16.445 bp for the godwit and 15.609 bp for the knot containing thirteen protein-coding genes, two rRNAs, twenty-two tRNAs, and a control region. The ATP8 and tRNA-Glu were not found in the knot. Bayesian phylogenetic analysis supported the position of both species in the clade of the Scolopacidae Family.

The Hudsonian godwit (*Limosa haemastica*) and the red knot (*Calidris canutus*) are two long-distance migratory shorebirds (Charadriiformes) which breed in the Arctic and spend the non-breeding season at high latitudes in the Southern Hemisphere (Hayman et al. 1986). The Hudsonian godwit is distributed in the Americas, with no subspecies described and breeds in three different areas: Alaska, Mackenzie Delta, and Hudson Bay (Walker et al. 2011). The main non-breeding areas are Chiloé Island (Chile), and bays along the Argentinian Patagonia and Tierra del Fuego (Senner et al. 2014). By contrast, the red knot has six subspecies described (*C.c. canutus*, *islandica*, *piersmai*, *rogersi*, *roselaari*, and *rufa*) that breeds along the Arctic of North America and Eurasia. Each subspecies migrates south to different non-breeding areas located across different continents (van Gils et al. 2020). Both species perform one of the longest migratory movements in the Animal kingdom. However, their populations are globally under a long-last declining (Runge et al. 2014) having both species high conservation interest, but specially the *C. c. rufa* subspecies (Baker et al. 2004).

DNA was extracted from blood samples of 10 godwits and 10 knots captured during Austral summer in two bays located in Chiloé Island (41°49′ S–73°37′ W and 42°28 S–73°41 W) using a commercial kit (E.Z.N.A Tissue kit DNA extraction, Omega Bio-Tek, USA) and later sequenced on a MiSeq®, Illumina (San Diego, CA, USA). Samples were deposited in the Facultad de Ciencias Veterinarias, Universidad Austral de Chile (UACH:Lhaemastica:1-10; and UACH:Ccanutus:1-10). Raw data (>9.5 million reads per species) was trimmed and filtered by quality using BBduk program. Reads were assembled and annotated in Geneious Prime (Biomatters Company, New Zealand) using *Limosa lapponica* (KX371106) and *Calidris tenuirostris* (MK992912) mitogenomes as references. A Bayesian phylogenetic tree was inferred with other 39 species Charadriiformes species using MrBayes 3.2.7 (Huelsenbeck and Ronquist 2001).

The mitochondrial genome of *L. haemastica* (Accession MT188757) and *C. canutus* (Accession MT183697) are 16.445 bp and 15.609 bp long, respectively, including both 13 protein-coding genes, 2 ribosomal RNA genes, 22 tRNA genes, and 1 control region (D-loop region). The base composition of each mitogenome was 30.5% for A, 29.9% for C, 14.1% for G, and 25.3% for T in *L. haemastica,* and 31% for A, 30.4% for C, 14.1% for G, and 24.5% for T in *C. canutus*. Overall, the genetic distance of *L. haemastica* was 9.6% with *L. lapponica*, and *C. canutus* was 9.5% with *C. tenuirostris*.

A gap of 117 bp in ND5 gene was found in the godwit when compared with *L. lapponica* (representing a 0.7% of the mitogenome) whereas a gap of 92 bp in COX1, 36 bp in COX2, and 350 bp in ND5, was found in *C. canutus* when compared with *C. tenuirostris*. Further, the ATP8 gene (243 bp) and the tRNA-Glu (∼76 bp) were not found in the knot. (representing a 5% of the mitogenome). At least two set of species-specific primers in the flanking regions for each gap were designed and resequenced by Sanger, but we were unable to fill those gaps. The reconstructed phylogeny supported the position of *L. haemastica* and *C. canutus* in the clade of the Scolopacidae family (Figure 1).

**Figure 1.**
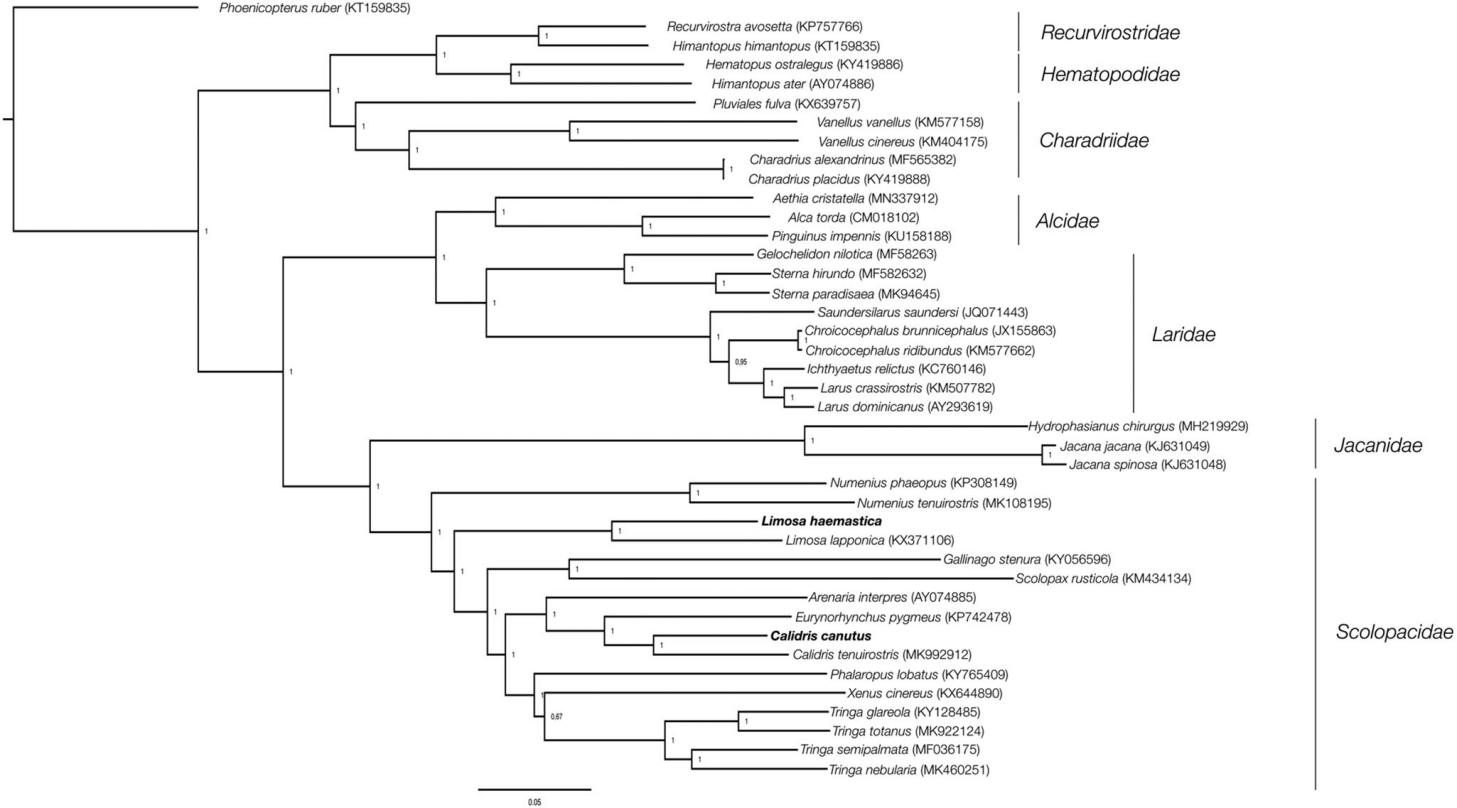
Bayesian phylogenetic tree inferred based on 15,211 nucleotides under GTR + I+G model. Posterior probabilities are included for each node.

The mitochondrial genome of two long-distance migratory shorebirds, *L. haemastica* and *C. canutus*, are reported which are relevant for further genetic studies on these threatened species.

## Data Availability

The data that support the findings of this study are opnely available in GenBank® at www.ncbi.nlm.nih.gov/genbank/, reference numbers MT188757 and MT183697.
